# Epidemiological trends and pathogen analysis of pediatric acute respiratory infections in Hanzhong Hospital, China: insights from 2023 to 2024

**DOI:** 10.3389/fpubh.2025.1557076

**Published:** 2025-04-25

**Authors:** Yuanfang He, Xiaoliang He, Ning He, Peipei Wang, You Gao, Jiexin Sheng, Jin Tang

**Affiliations:** ^1^Department of Clinical Laboratory, Hanzhong Central Hospital, Hanzhong, Shaanxi, China; ^2^Department of Urology, Hanzhong Central Hospital, Hanzhong, Shaanxi, China; ^3^Department of Radiology, Hanzhong Central Hospital, Hanzhong, Shaanxi, China

**Keywords:** children, acute respiratory infection, epidemiology, respiratory pathogens, co-infection

## Abstract

**Background:**

Acute respiratory infections (ARIs) are a leading cause of morbidity in children. Understanding the distribution of respiratory pathogens is crucial for effective prevention and treatment. This study analyzed the prevalence and epidemiological characteristics of ARIs in children from 2023 to 2024.

**Methodology:**

This study included 9,294 children aged 0–18 years with ARI symptoms who were treated between July 2023 and August 2024. Respiratory samples were tested using polymerase chain reaction (PCR) for eight common viruses. Data were analyzed by age and gender to assess pathogen distribution and demographic patterns.

**Results:**

A total of 14,722 samples were enrolled, with 2,888 (19.62%) testing positive for at least one pathogen. Among these, single-pathogen infections were predominant (97.66%, 2,756/2,822), while co-infections were less frequent (2.34%, 66/2,822). The three most common pathogens were adenovirus (ADV, 33.24%), *Mycoplasma pneumoniae* (MP, 25.07%), and whooping cough (WC, 22.78%). No significant gender differences were observed in the overall positive rate or pathogen distribution (*p* > 0.05). Pathogen detection rates varied significantly by age group (*χ*^2^ = 110.03, *p* < 0.001), with WC and MP being most prevalent in school-age children (*χ*^2^ = 104.58, *p* < 0.001; *χ*^2^ = 11.546, *p* = 0.009, respectively) and Flu A more frequent in preschool children (*χ*^2^ = 38.738, *p* < 0.001). MP, WC, ADV, human rhinovirus, and human metapneumovirus were detected throughout the year.

**Discussion:**

The findings highlight that ARIs in children are primarily caused by single respiratory pathogens, with significant age-related differences in pathogen prevalence. These results emphasize the need for age-specific prevention strategies, such as targeted vaccination programs and public health interventions, particularly for school-age children during peak transmission periods.

## Introduction

Acute respiratory infections (ARIs) are among the most prevalent and burdensome diseases affecting children worldwide, contributing significantly to morbidity and mortality (1). Globally, ARIs are responsible for more deaths than tuberculosis and acquired immune deficiency syndrome combined (2). The primary pathogens include adenovirus (ADV), mycoplasma pneumonia (MP), influenza virus (Flu), and human rhinovirus (HRV), which collectively impose a heavy burden on healthcare systems (3). In children, ARIs manifest with overlapping symptoms such as fever, cough, and nasal congestion, complicating diagnosis and delaying appropriate treatment (4). Accurate detection of viruses is pivotal for early isolation and appropriate treatment, reducing the overuse of antibiotics in cases where pathogens remain unclear.

Despite the global impact of ARIs, the epidemiological patterns of respiratory viruses in different regions vary significantly. Studies from high-income countries, such as the United States and the United Kingdom, report that respiratory syncytial virus (RSV) and influenza viruses account for approximately 30–40% of pediatric hospitalizations due to respiratory illnesses (5, 6). In contrast, China exhibits substantial regional differences in respiratory virus prevalence. For example, studies from Beijing and Shanghai indicate RSV positivity rates of 25–30%, while research from Wuhan and other central provinces reports lower detection rates, ranging from 15 to 22% (7, 8). Similarly, ADV has been reported with positivity rates of 5–10% in developed areas but up to 12–15% in rural regions (9, 10).

Previous studies on respiratory pathogens in China have predominantly focused on urban centers, where healthcare infrastructure and diagnostic technologies are more advanced. For instance, research from 2019 highlighted the high detection rates of respiratory pathogens in hospitalized children with lower respiratory tract infections but was constrained by its reliance on nasopharyngeal specimens, limiting subtype-specific epidemiological analyses for pathogens like parainfluenza virus type 3 and HRV species C (11, 12). Similarly, a 2018 study in central Fujian identified HRV, influenza A, and human ADV as the most common pathogens. Still, its findings were limited by a small sample size and the relatively developed context of the region (13).The COVID-19 pandemic further altered respiratory virus transmission dynamics due to widespread non-pharmacological interventions such as mask-wearing and social distancing (14). While these measures likely influenced the prevalence and transmission of respiratory pathogens, their long-term impact, particularly on pediatric populations in semi-urban and rural settings, remains unclear. Hanzhong, a semi-urban area in western China, presents unique epidemiological characteristics that have been underrepresented in existing research. Hanzhong has a population of approximately 3.21 million, with an urbanization rate of 53%, which is lower than the national average. This reflects its significant rural population, creating disparities in healthcare access compared to more urbanized regions of China. Unlike major cities like Beijing or Shanghai, which have higher urbanization rates and more evenly distributed healthcare resources, Hanzhong faces challenges such as limited access to healthcare in rural areas, fewer medical professionals, and underdeveloped diagnostic infrastructure. By focusing on Hanzhong Central Hospital, a tertiary care facility that serves both urban and surrounding rural populations, this study seeks to fill a critical gap in understanding respiratory infections in underrepresented regions, where healthcare access may be more limited. This is especially timely, as the COVID-19 pandemic has reshaped the epidemiology of respiratory pathogens, making it crucial to understand how these changes affect semi-urban and rural populations for effective public health interventions.

Beyond its health implications, ARIs also impose significant economic burdens. In developed countries, direct medical expenses for pediatric respiratory infections, including hospitalization and outpatient visits, exceed billions of dollars annually (15). Indirect costs, such as parental work absences and lost productivity, further contribute to the economic strain. A study in the United States estimated that parental absences due to child respiratory illnesses result in annual productivity losses exceeding $2 billion (16, 17). In China, where healthcare access disparities persist between urban and rural areas, the financial burden on families in less developed regions can be even more pronounced. Therefore, understanding ARI epidemiology in underdeveloped areas is crucial for formulating cost-effective prevention and intervention strategies.

The primary objective of this study was to determine the prevalence and distribution of eight common respiratory pathogens among children diagnosed with ARIs at Hanzhong Central Hospital between July 2023 and August 2024. In addition to estimating overall pathogen prevalence, the study aimed to evaluate age-related differences in positivity rates, explore gender-based variations in pathogen distribution, and identify seasonal trends in detection. Furthermore, the analysis sought to characterize the frequency and composition of mixed infections involving two or more pathogens, thereby providing a more comprehensive understanding of ARI epidemiology in a semi-urban pediatric population. Utilizing nucleic acid-based detection methods, it systematically examines viral prevalence and epidemiological trends across different age and gender groups. The findings aim to provide clinicians and public health authorities with valuable insights for developing evidence-based diagnostic tools and prevention strategies tailored to the specific needs of underdeveloped small-town settings.

## Methods

### Study design and setting

This study employed a retrospective design to investigate children diagnosed with ARIs who were treated at Hanzhong Central Hospital between July 2023 and August 2024. Eligible participants were children under 18 years of age who met the diagnostic criteria for ARIs, defined as any case with the sudden onset of symptoms such as fever, cough, sore throat, nasal congestion, and difficulty breathing (18). Exclusion criteria included children with non-infectious respiratory conditions, such as congenital heart disease or chronic lung disease, and those with healthcare-associated infections. The study categorized participants into four age groups for analysis, namely, infants (<1 year), toddlers (1 to <3 years), preschoolers (3 to <6 years), and school-age children (6 to <18 years). This retrospective study was approved by the Medical Ethics Committee of Hanzhong Central Hospital and met the requirements for exemption from informed consent.

### Sample collection and pathogen detection

Nasopharyngeal swab specimens were collected from all participants at the time of clinical presentation. Samples were placed into sterile viral transport media and transported under cold-chain conditions (2–8°C) to the laboratory for processing within 6 h. Laboratory testing targeted eight common respiratory pathogens, including influenza A virus (Flu A), influenza B virus (Flu B), ADV, MP, respiratory syncytial virus (RSV), HRV, human metapneumovirus (hMPV), and whooping cough (WC). Nucleic acid extraction was conducted using the Tianlong automatic nucleic acid extractor (Tianlong Science and Technology Co., Ltd., Xi’an, China). All target pathogens were detected using polymerase chain reaction (PCR). MP and RSV were amplified using the AFD4800 Real-Time PCR System (Anjess Medical Technology Co., Ltd., Hangzhou, China), while Flu A, Flu B, HPV, HRV, ADV, and BP were amplified using the MA-6000 Real-Time PCR System (Yarui Biotechnology Co., Ltd., Suzhou, China). The thermal cycling conditions varied depending on the pathogen. For MP, the reaction was performed with an initial denaturation at 93°C for 2 min, followed by 10 cycles of denaturation at 93°C for 45 s and annealing at 55°C for 60 s, then 30 additional cycles of 93°C for 30 s and 55°C for 45 s with fluorescence signal collection. For Flu A and Flu B, the reaction included an initial step at 50°C for 15 min and 95°C for 15 min, followed by 45 cycles of 94°C for 15 s and 58°C for 45 s. For BP and ADV, the protocol started with an initial step at 50°C for 2 min and 94°C for 5 min, followed by 45 cycles of 94°C for 15 s and 57°C for 30 s, with a final extension at 25°C for 10 s. For RSV, the cycling conditions included an initial step at 40°C for 30 min and 94°C for 3 min, followed by 10 cycles of 93°C for 15 s and 55°C for 45 s, then 30 additional cycles of 93°C for 15 s and 55°C for 45 s. For HRV and HPIV, the reaction was performed with an initial step at 50°C for 15 min and 95°C for 15 min, followed by 40 cycles of 94°C for 15 s and 55°C for 45 s, with a final extension at 40°C for 20 s. Commercial nucleic acid detection kits were used for pathogen identification, with kits for Flu A, Flu B, HRV, HPIV, MP, and RSV obtained from Guangzhou Daan Gene Co., Ltd., while kits for BP and ADV were obtained from Hunan Shengxiang Biotechnology Co., Ltd. Negative and positive controls were included in every PCR run to ensure accuracy and reliability.

### Quality control

Positive and negative controls were included in each PCR run to ensure the validity of results. Only results that passed quality control standards were reported, while failed tests were repeated after troubleshooting and, if necessary, re-sampling.

### Outcome measures

To reduce selection bias, we included all eligible pediatric patients diagnosed with ARIs during the study period without restriction based on severity or treatment outcome. Information bias was minimized by relying on standardized diagnostic criteria and laboratory protocols across all cases. The primary outcome was the overall detection rate of respiratory pathogens, defined as the proportion of children testing positive for at least one pathogen among the total tested. Secondary outcomes included pathogen detection rates stratified by age group and gender, as well as seasonal variations. Raw data were systematically reviewed for errors, duplicates, and missing values. Duplicated entries were removed, and incomplete records were excluded if they lacked key variables necessary for the analysis. In cases where a patient underwent repeated testing for the same pathogen within a short period, only the first sampling result was included in the final analysis. Subsequent tests conducted within the same clinical episode were excluded to avoid duplication. Patient records were systematically reviewed to identify and remove duplicate entries before statistical analysis, ensuring that each case was counted only once. No missing data were present in any key variables, ensuring high data integrity.

### Statistical analysis

Data were analyzed using SPSS version 29.0 (IBM, Armonk, NY). Demographic and clinical characteristics were summarized using descriptive statistics: continuous variables were reported as means ± standard deviations, while categorical variables were presented as frequencies and percentages. Statistical comparisons between groups were conducted using chi-square tests or Fisher’s exact tests as appropriate. Logistic regression analysis was performed to assess associations between demographic variables and pathogen detection, with adjustments for potential confounding factors. Confounders were included based on their clinical relevance and statistical significance in univariable analysis (*p*-value <0.10). Seasonal trends in pathogen prevalence were examined through time-series analysis to evaluate monthly variations. Statistical significance was set at a two-tailed *p*-value <0.05. To account for type I errors in multiple comparisons, Bonferroni correction was applied. All analyses were independently reviewed by a second analyst to ensure reproducibility and accuracy.

## Results

### Detection and distribution of respiratory pathogens in children

**Figure 1:**
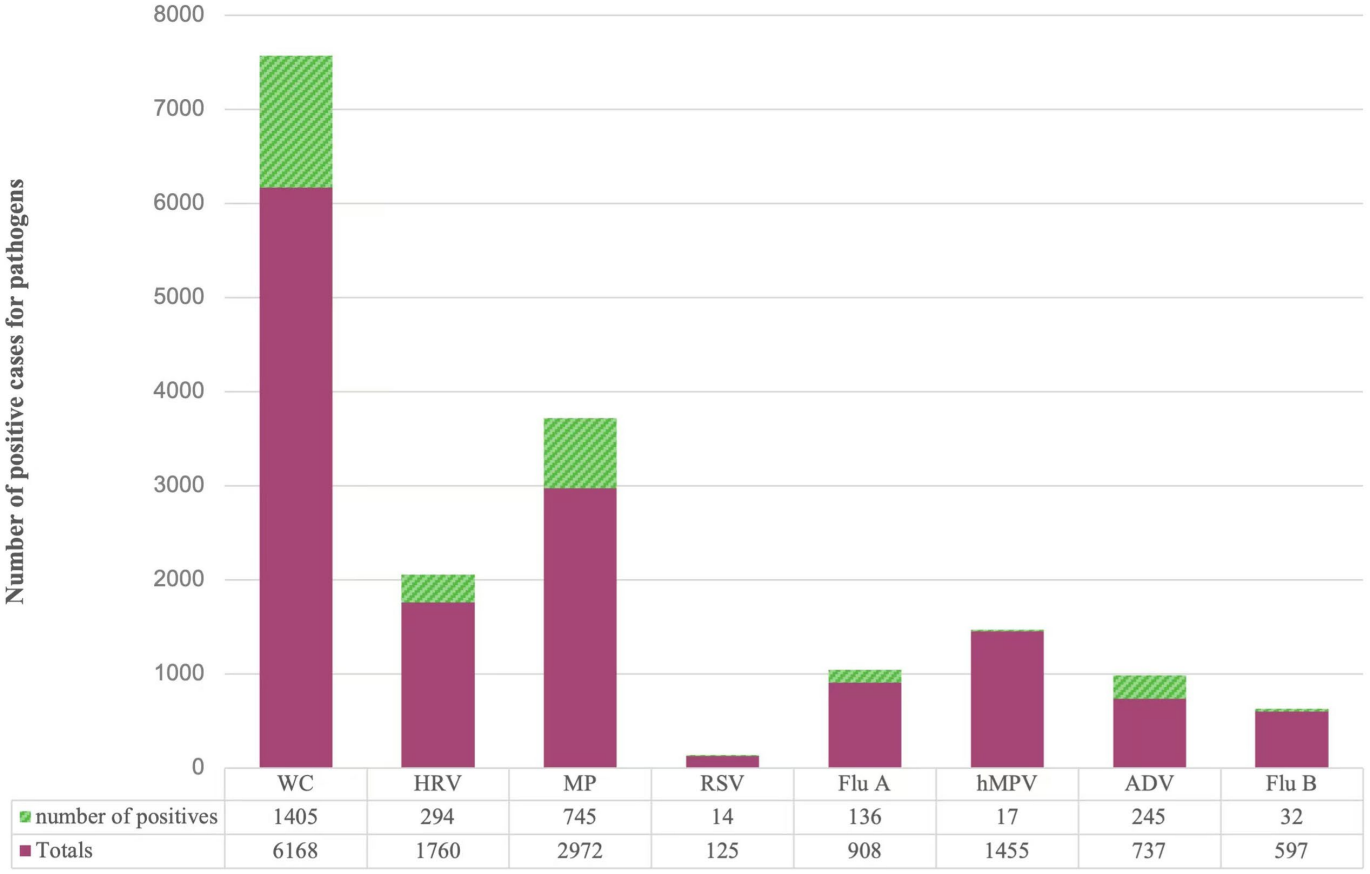
Distribution of different pathogens detected in children with acute respiratory disease. WC: whooping cough; HRV: rhinovirus; MP: Mycoplasma pneumoniae; RSV: respiratory syncytial virus; Flu A: influenza A virus; hMPV: human metapneumovirus; ADV: adenovirus; Flu B: influenza B virus.

A total of 14,722 samples were included in this study, and pathogens were detected in 2888 (19.62%) cases. Among the 8 respiratory pathogens, the pathogens with higher detection rates were ADV (33.24%), MP (25.07%), WC (22.78%), HRV (16.70%), Flu A (14.98%), RSV (11.20%), Flu B (5.36%), and hMPV (1.17%) in the order of 8 respiratory pathogens. The situation is detailed in Figure 1.

### Detection of respiratory pathogens by gender

Among the 7,883 male samples, 1,557 cases were detected positively, and among the 6,839 female samples, 1,331 cases were detected positively. The difference in the detection rates of the eight respiratory pathogens among children of different genders was not statistically significant (*p* > 0.05), and the difference in the total detection rate of respiratory pathogens among different genders was not statistically significant (*p* > 0.05), as shown in Table 1.

**Table 1:** Detection of respiratory viruses by gender.

variables	WC	HRV	MP	RSV	Flu A	hMPV	ADV	Flu B	Total positive rate
Male, *n* (%)	733(22.57)	166(17.20)	406(25.62)	6(10.17)	83(16.63)	11(1.35)	134(34.72)	18(5.52)	1,557(19.75)
Female, *n* (%)	672(23.01)	128(16.10)	339(24.44)	8(12.12)	53(12.96)	6(0.94)	111(31.62)	14(5.17)	1,331(19.46)
*χ*^2^ value	0.174	0.380	0.543	0.119	2.384	0.527	0.792	0.037	0.195
*P*-value	0.677	0.538	0.461	0.730	0.123	0.468	0.374	0.848	0.659

WC, whooping cough; HRV, rhinovirus; MP, *Mycoplasma pneumoniae*; RSV, respiratory syncytial virus; Flu A, influenza A virus; hMPV, human metapneumovirus; ADV, adenovirus; Flu B, influenza B virus.

### Detection of respiratory pathogens at different ages

After comparing the distribution of respiratory pathogens in 2888 samples which tested positive for respiratory pathogens according to age groups, the total detection rate of respiratory pathogens was the highest in the school-age group (*χ^2^* = 110.03, *p* < 0.001), and the difference between different age groups was statistically significant, and the positive rate gradually increased with the increase of age, as shown in Table 2 (*χ^2^* = 104.58, *p* < 0.001), while Flu A was mostly found in the preschool group (*χ^2^* = 38.738, *p* < 0.001). MP had the highest positive rate in the school-age group (*χ^2^* = 11.546, *p* = 0.009), and the positive rate gradually increased with age. hMPV was mostly found in the infant group (6.00%, 3/50), and the positive rate decreased with age. Decreased, whereas ADV, on the contrary, was mostly seen in the school-age group (42.31%, 11/26), and the positivity rate increased with age.

**Table 2:** Detection of respiratory viruses in different ages.

Variables	WC	HRV	MP	RSV	Flu A	hMPV	ADV	Flu B	Total positive rate
Infants, *n* (%)	32(17.20)	10(20.41)	3(9.09)	4(36.36)	0(0)	3(6.00)	1(16.67)	0(0)	53(13.09)
Toddlers, *n* (%)	855(21.40)	255(16.35)	410(23.50)	9(9.28)	47(11.44)	14(1.08)	201(32.21)	15(5.34)	1806(18.05)
Preschool, *n* (%)	164(17.10)	17(22.08)	254(27.79)	1(11.11)	84(23.08)	0(0)	32(39.51)	14(6.25)	566(21.10)
School-age, *n* (%)	354(34.44)	12(16.22)	78(27.86)	0(0)	5(5.68)	0(0)	11(42.31)	3(4.48)	463(28.40)
*χ*^2^-value	104.58	2.238	11.546	6.320	38.738	6.598	3.336	1.164	110.03
*P*-value	<0.001	0.525	0.009	0.061	<0.001	0.066	0.347	0.761	<0.001

WC, whooping cough; HRV, rhinovirus; MP, *Mycoplasma pneumoniae*; RSV, respiratory syncytial virus; Flu A, influenza A virus; hMPV, human metapneumovirus; ADV, adenovirus; Flu B, influenza B virus.

### Detection of respiratory pathogens in different months

**Figure 2:**
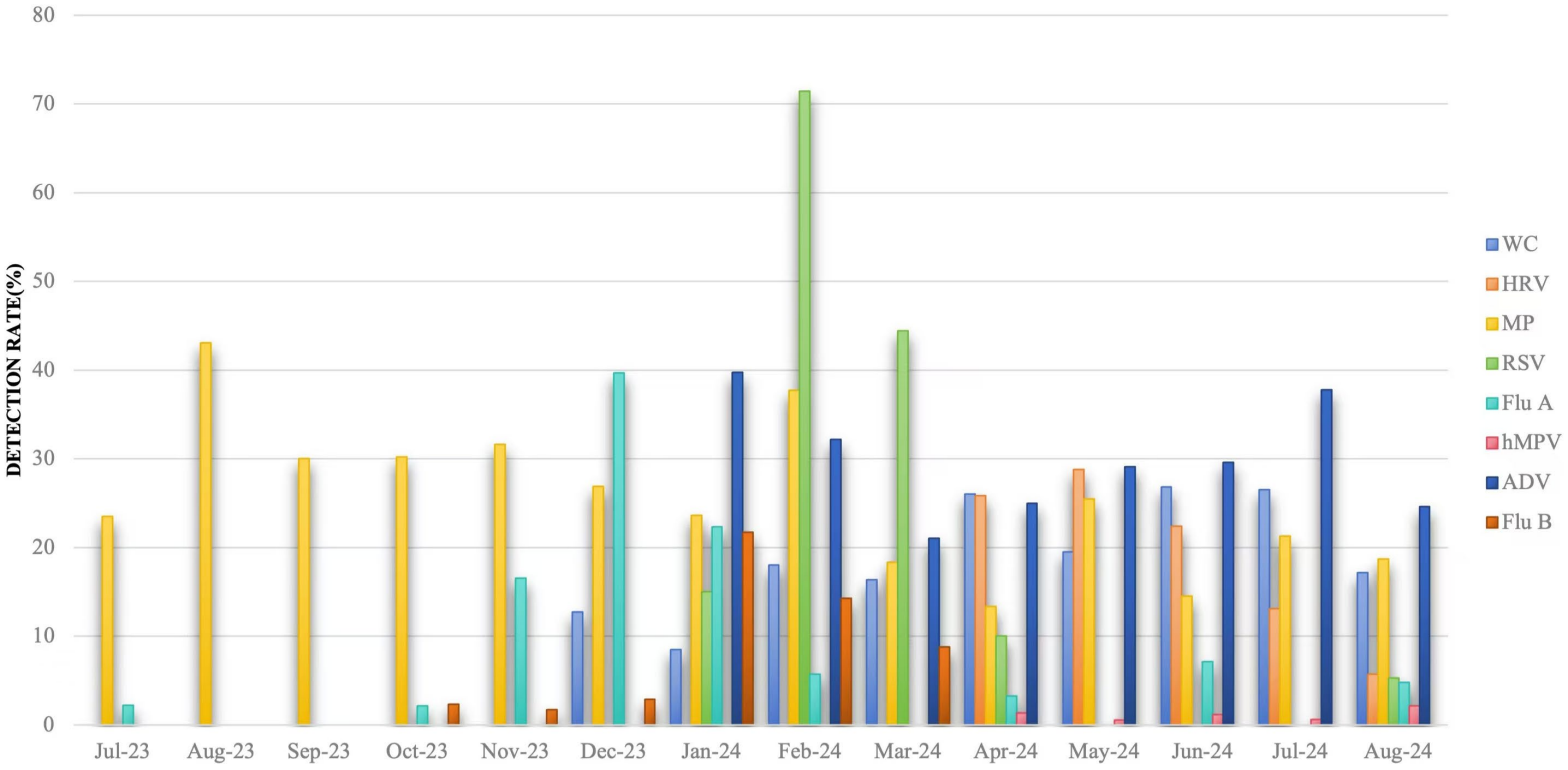
Detection rate of multiple respiratory pathogens in different months. Jul: July; Aug: August; Sep: September; Oct: October; Nov: November; Dec: December; Jan: January; Feb: February; Mar: March; Apr: April; Jun: June. WC: whooping cough; HRV: rhinovirus; MP: Mycoplasma pneumoniae; RSV: respiratory syncytial virus; Flu A: influenza A virus; hMPV: human metapneumovirus; ADV: adenovirus; Flu B: influenza B virus.

Between July 2023 and December 2023, MP had a higher detection rate of 43.09% in August 2023, Flu A had a higher detection rate of 39.70% in December 2023, and the rest of the months were lower. Between January 2024 and August 2024, the detection rate of ADV was consistently high, with a mean value of 29.88%, and the detection rates of Flu A and Flu B were higher in January 2024, with 22.34 and 21.69%, respectively, was higher in January 2024 with 22.34 and 21.69%, respectively, MP was higher in February 2024 with 37.68%, WC was higher in April, June and July 2024 with 26.02, 26.83, and 26.49%, respectively, and lower in remaining months, as shown in Figure 2.

### Distribution of mixed detected respiratory pathogens

A total of 9,294 children were included in this study, of which 2,822 tested positive, 97.66%, 2,756/2,822 were mono-infected and 2.34%, 66/2,822 were multi-infected. A total of 66 children were detected with mixed infections of respiratory pathogens, and all of them were mixed infections of 2 pathogens. Further analysis revealed that WC combined with HRV was the most common pathogen in mixed infections, followed by WC combined with MP mixed infections, and the distribution of other pathogens is detailed in Table 3.

**Table 3:** Distribution of specific pathogens in 66 children with mixed detection of respiratory pathogens.

Pathogens	WC	HRV	MP	RSV	Flu A	hMPV	ADV
HRV	24						
MP	18	10					
RSV	/	/	1				
Flu A	1	/	1	/			
hMPV	/	1	/	/	/		
ADV	1	1	5	/	/	/	
Flu B	/	/	/	/	1	/	2

## Discussion

The ARIs pose a significant global health challenge, particularly among children in developing regions, where they account for substantial morbidity and mortality (19). This study provides valuable insights into the prevalence and epidemiological characteristics of respiratory pathogens in children from underdeveloped small-town areas, addressing a critical gap in regional surveillance data and offering actionable information for targeted prevention and control strategies.

The positivity rate for the eight respiratory pathogens examined was 19.62%, lower than rates reported in other countries but slightly higher than the advanced city, Suzhou in China (17.19%) (9, 20). Studies from other cities in China, such as Beijing and Wuhan, report higher positivity rates for respiratory pathogens, with values reaching 23.4 and 22.1%, respectively (21, 22). Among these pathogens, ADV demonstrated the highest positivity rate (33.24%), surpassing MP (25.07%). These findings differ from those in Shenzhen, where MP exhibited higher positivity rates than ADV (23). Such differences may be attributed to factors such as regional healthcare infrastructure, population density, and environmental conditions. Additionally, varying diagnostic protocols, sample collection methods, and monitoring timelines across regions could contribute to the observed discrepancies (24, 25). This highlights the importance of considering regional factors when interpreting epidemiological data, as they may significantly influence the detection rates of respiratory pathogens.

Our study observed no significant gender-based differences in the detection rates of the eight respiratory pathogens. However, previous research has suggested that males are often at higher risk of morbidity and mortality from respiratory infections, including influenza (26, 27). This study’s absence of such differences could be due to the limited monitoring period or narrower testing scope, underscoring the need for further investigation.

Age-related susceptibility to respiratory pathogens was evident in this study. School-age children exhibited the highest detection rates, with positivity increasing with age. This discrepancy may stem from differences in immune protection, where younger children benefit from maternal antibodies, which gradually wane over time (28, 29). In contrast, school-age children experience increased exposure in dense classroom settings with limited disease mitigation measures, a factor that has been consistently highlighted in epidemiological research on respiratory infections (30, 31). These findings underscore the importance of enhancing disease surveillance and health education among school-age populations to mitigate transmission risks. A study demonstrated that targeted interventions, such as improved classroom ventilation, regular hand hygiene, and vaccination campaigns, significantly reduce the spread of respiratory infections in school settings (32). Incorporating these strategies into public health policies could help reduce the burden of respiratory diseases in this high-risk population.

Among the pathogens detected, MP and ADV were the most prevalent, with ADV positivity increasing with age, consistent with findings by Sun et al. (9). The higher prevalence of ADV in older children may be due to a combination of factors, including cumulative exposure in school settings, where respiratory pathogens spread more easily, and the development of immunity over time, which might offer more protection to younger children (33). Conversely, MP was notably prevalent in younger children, particularly infants and toddlers, which may reflect their underdeveloped immune systems and increased vulnerability to bacterial infections (34). Age-related variations in pathogen prevalence have been reported globally, with similar trends observed in studies from other regions (35, 36). Additionally, regional and subtype-specific variations in the distribution of respiratory pathogens may contribute to these differences. For example, ADV has been found to exhibit seasonal peaks in certain countries, with increased circulation during the colder months, which could explain its higher detection rates in older children who have been exposed to these seasonal fluctuations. These age-related patterns likely reflect regional and subtype-specific variations. WC was notably more prevalent among school-age children, mirroring national and global trends of rising pertussis incidence (37). These findings highlight the importance of maintaining robust pertussis vaccination and surveillance programs to control its spread.

Mixed infections were identified in 2.34% of cases, primarily involving combinations of two pathogens, with WC combined with HRV and WC combined with MP being the most common. This rate is lower than reported in other countries, reflecting regional variations in co-infection prevalence and pathogen susceptibility (38, 39). The presence of mixed infections underscores the complexity of ARIs and the need for comprehensive diagnostic approaches to guide effective treatment strategies.

This study provides valuable insights into the prevalence and epidemiological characteristics of respiratory pathogens among children in underdeveloped small-town areas. It addresses a critical gap in regional surveillance data and offers actionable information for developing targeted prevention and control strategies. Several limitations should be acknowledged. First, it is a retrospective study based on single-center nasopharyngeal swab specimens, which may carry a risk of underdetection. Incorporating bacterial culture and serological testing in future studies could address this limitation and improve diagnostic accuracy. Additionally, the temporal scope was restricted, as testing for ADV only began in early 2024, limiting the generalizability of the findings. Extending the monitoring period in future research would help capture long-term epidemiological trends more comprehensively.

The results of this study offer several important implications for public health policy in China. First, the observed differences in pathogen prevalence across age groups and seasons underscore the need for region-specific surveillance and early warning systems, particularly in semi-urban and rural areas. The high prevalence of adenovirus and *Mycoplasma pneumoniae* highlights the importance of multiplex PCR testing in routine diagnostics to enable timely identification and management of outbreaks. Additionally, given the disparities in healthcare access between urban and rural areas, there is a need to strengthen primary care infrastructure and laboratory diagnostic capacity in underdeveloped regions. These findings provide actionable evidence to support more equitable and targeted respiratory infection control strategies at both regional and national levels.

This study lays the groundwork for future investigations into pediatric respiratory infections in underrepresented semi-urban and rural settings. Given the study’s single-center and limited temporal scope, future research should include multi-center, multi-year surveillance to validate the generalizability of these findings and better capture long-term seasonal and epidemiological trends. Expanding the diagnostic spectrum to include bacterial and fungal pathogens, as well as implementing serological testing, would further enhance the accuracy and comprehensiveness of pathogen identification. Additionally, integrating clinical outcome data—such as hospitalization rates, treatment responses, and recovery times—could help link specific pathogens to disease severity, informing both clinical management and policy development. Finally, evaluating the impact of public health interventions (e.g., school-based hygiene programs or vaccination campaigns) through prospective studies could provide evidence for scalable prevention strategies in resource-limited settings.

## Conclusion

This study identified ADV, MP, and WC as the predominant respiratory pathogens among children with ARIs in underdeveloped small-town areas, with significant age-related differences in pathogen prevalence. These findings contribute to the epidemiological understanding of ARIs in such regions and underscore the necessity of systematic, long-term surveillance to inform effective public health interventions. By enhancing pathogen monitoring and strengthening prevention strategies, particularly for school-age children, health outcomes in this vulnerable population can be substantially improved.

## Data Availability

The original contributions presented in the study are included in the article/supplementary material, further inquiries can be directed to the corresponding author.
